# Identifying anthropogenic features at Seoke (Botswana) using pXRF: Expanding the record of southern African Stone Walled Sites

**DOI:** 10.1371/journal.pone.0250776

**Published:** 2021-05-12

**Authors:** Stefano Biagetti, Jonas Alcaina-Mateos, Abel Ruiz-Giralt, Carla Lancelotti, Patricia Groenewald, Jordi Ibañez-Insa, Shira Gur-Arie, Fred Morton, Stefania Merlo

**Affiliations:** 1 CaSEs Research Group, Department of Humanities, Universitat Pompeu Fabra, IMF-CSIC, Barcelona, Spain; 2 School of Geography, Archaeology and Environmental Studies (GAES), University of the Witwatersrand, Johannesburg, South Africa; 3 ICREA, Passeig Lluís Companys 23, Barcelona, Spain; 4 Department of Archaeology, University of Cape Town, Cape Town, South Africa; 5 Geosciences Barcelona (GEO3BCN), CSIC, Barcelona, Spain; 6 Department of Maritime Civilizations, University of Haifa, Haifa, Israel; 7 Department of History, University of Botswana, Gaborone, Botswana; 8 McDonald Institute for Archaeological Research, University of Cambridge, Cambridge, United Kingdom; University of Western Australia, AUSTRALIA

## Abstract

Numerous and extensive ‘Stone Walled Sites’ have been identified in southern African Iron Age landscapes. Appearing from around 1200 CE, and showing considerable variability in size and form, these settlements are named after the dry-stone wall structures that characterize them. Stone Walled Sites were occupied by various Bantu-speaking agropastoral communities. In this paper we test the use of pXRF (portable X-ray fluorescence analysis) to generate a ‘supplementary’ archaeological record where evident stratigraphy is lacking, survey conditions may be uneven, and excavations limited, due to the overall site size. We propose herein the application of portable X-ray fluorescence analysis (pXRF) coupled with multivariate exploratory analysis and geostatistical modelling at Seoke, a southern African SWS of historical age (18^th^ century CE). The aim of the paper is twofold: to explore the potential of the application of a low cost, quick, and minimally invasive technique to detect chemical markers in anthropogenic sediments from a Stone Walled Site, and to propose a way to analyse the results in order to improve our understanding of the use of space at non-generalized scales in such sites.

## Introduction

**Fig 1:**
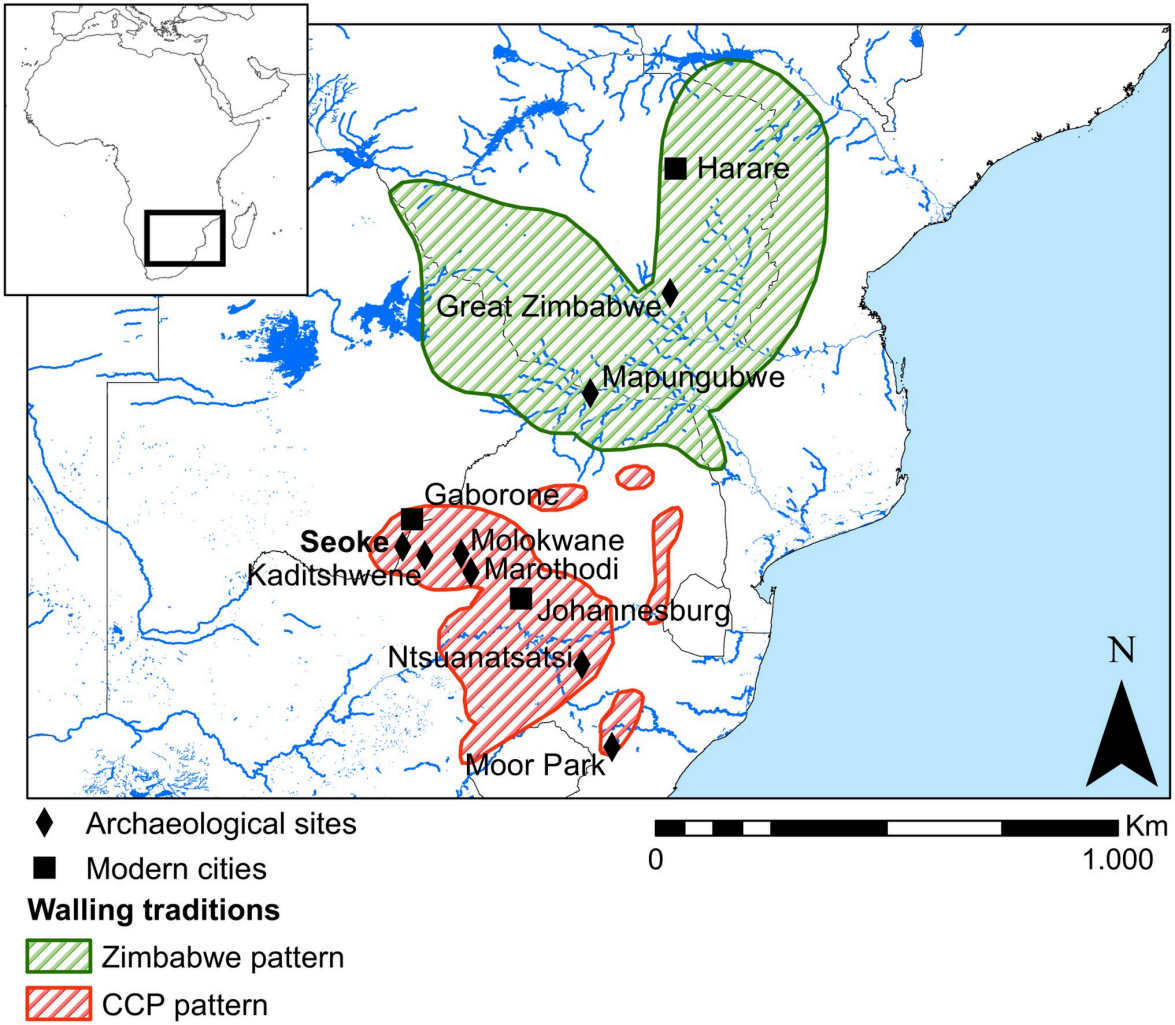
Distribution of Stone Walled sites. Map showing the distribution of stone-wall settlements in the second millennium CE in southern Africa (adapted from [1]).

Numerous and extensive ‘Stone Walled Sites’ are attested in the whole of southern Africa between the Orange and the Zambezi rivers (Fig 1). Appearing from around 1200CE, and showing considerable variability in size and form, these settlements are named after the dry-stone wall structures that characterize them. Stone Walled Sites (SWS) were occupied by various Bantu-speaking agropastoral communities who cultivated crops, hunted, and venerated cattle as the source of both economic and political wealth [1].

Archaeologists have long focused on the study of such sites [2–5] whose size and visibility make them relatively easy to recognise in the landscape, in particular through aerial photographs [4, 6–8] and, more recently, satellite imagery [9]. Two broad stone walling traditions have been identified in southern Africa, encompassing a vast area that includes present day Zimbabwe, Zambia, Mozambique, Botswana and South Africa: these are commonly known as the Zimbabwe Pattern, which spread in the northern part of southern Africa and is first attested at Mapungubwe around 1250 CE, and the Central Cattle Pattern (CCP) [1] to which the site of Seoke can be ascribed. Contrarily to what is believed for the Zimbabwe tradition where the walling is considered to have been a marker of class differences used for the seclusion of elites’ spaces and ritual practices [1], the stone walls in the CCP tradition were not exclusive to elite spaces and they helped to separate cattle from people, household from household, and the entire settlement from its surroundings [1]. The earliest known example of the CCP pattern, which is spread over the southern part of the region, is Moor Park in Kwa-Zulu Natal and dates from the thirteenth to the fifteenth century [1]. This early walling is associated with Nguni speakers. Sotho-Tswana stone walled sites are associated within later clusters, called N, V and Z by Maggs [7]. The oldest known walling of this cluster occurs near the hill of Ntsuanatsatsi in the Free State province of South Africa and dates to around 1500 CE. Ascribed to the CCP pattern, a series of large-scale Tswana towns in the area between the Pilanesberg/Magaliesberg and southern Botswana are considered the expression of the extensive movement of Tswana groups from the Highveld into the Kalahari. Developing in the mid-1700s and reaching their ultimate expressions by the early 19^th^ century, these extensive stone walled sites—typically associated with late Moloko ceramics—were the capitals of aggregated Tswana-speaking communities—entire chiefdoms living together in a single town under the authority of their resident ruler [10–17]. The considerable size of the largest of these towns, reaching between 10,000 and 20,000, inspired the term ‘mega-sites’ in earlier archaeological literature [18]. Their density and scale bear testimony to significant changes that were underway in southern Africa during this period. Notwithstanding the long tradition of research around the use of space in these settlements [1, 19, 20], based principally on ethnographic evidence and excavation of limited portions of exemplar sites, SWS of the CCP tradition are difficult to tackle with traditional approaches beyond a general architectonic assessment. The sites were often occupied for short periods of time (usually one or two generations) and are characterized by (i) very thin archaeological deposits, (ii) scarcity of artefacts in most of the deposits and/or on the surface, and (iii) a large number of stone structures with similar morphology or other macroscopic physical characteristics, making it impossible to identify their diverse use during site life, in particular beyond the areas of the cattle enclosures and the main dwellings. Traditionally, excavations have focused on middens and stock enclosures, the most evident and culturally “rich” deposits, to extract datable artefacts and construct building chronological sequences. The difficulties in retrieving a broader archaeological record coupled with the relative late chronology of such sites have prompted the adoption of the so-called ‘Direct Historical Approach’, a methodology developed in the US during the 1920s-1930s, which argued that knowledge relating to historical and recent periods is extended back into earlier times. Archaeological research on Tswana towns has, since the 1980s, predominantly revolved around the application of an ethnographically derived normative model, known as the ‘Central Cattle Pattern’, for the interpretation of settlement space and organisation [19–22]. This model explains the settlement pattern through a structural approach, where cattle are positioned at the centre of the settlement and there is a distinct division between male and female occupation of space. Huffman has emphasised that a normative model is useful in order to gain insight in generalised aspects of a society and its organisational principles, but that it is not useful for investigating the details of daily behaviour and dynamics [22]. It is also not concerned with variation among the group identities since it subsumes subtle differences to extract common underlying principles. As such “To understand the meaning of variations within the Central Cattle Pattern, it is necessary to construct models at a lower, less general scale.” [22, p.24].

The large number of papers published in the last 30 years testifies to the influence of this interpretive framework. Yet, many issues have been raised [23–28] related to the application of ‘ethnographic reports’ mostly collected during the colonial period, and by using an ‘ethnographic present’ restricted to some specific societies (Eastern Bantu speaking group [22]). Complicating the picture is the heterogeneous nature of Tswana societies, which typically included diverse ethnicities [14, 15]. To this end, an alternative approach to the understanding of the functional and symbolic use of space at different scales at SWS may focus on the anthropogenic deposits per se. Anthropogenic deposits often represent the main component of archaeological sites and are a primary source of information on the human activities carried out in the past. Human occupations may leave evidence in the form of chemical elements in the archaeological sediments [29]. Since the seminal works of Barba and Ortiz [30] and Middleton and Price [31], the study of such elements has increasingly been applied in archaeology [32, and references therein] and ethnoarchaeology to identify the chemical signatures of many human activities (e.g. food preparation and consumption, craft production, livestock management) [33]. Whilst these approaches have had a limited use in an African context, their application to study both vertical [34, 35] and horizontal [36–39] anthropic traces has proven to be successful.

Chemical markers represent an invaluable approach to pin down past and recent activities at a site, to understand the spatial dynamics of such activities, and to interpret architectural structures in relation to their functions. The potential of this approach resides in that the chemical elements’ signatures represent the repetitive use of a determined space and that are little affected by datable events [40]. Under this approach, the focus shifts from the absolute values of the chemical elements to their presence, combination and, especially, the anomalies created by their deviation from the average of the samples. Consequently, the anthropogenic signature of spaces is not site-dependent and results are comparable across widely different geographical and chronological contexts. For instance, major components of organic occupation waste are Ca, Sr, K, P, Mn and Zn since they are essential nutrients for all living organisms, and their occurrence can be linked to specific features and activities to be found at archaeological sites. Recent reviews [32, 41, 42] have identified the significant relationships between chemical elements and archaeological features, such as burials, hearths, stock enclosures, middens, houses, metal working areas, and food-processing areas. As stressed also by Save and colleagues [32], “[‥] geochemistry has experienced an increase in interest from archaeologists in search of new methods to investigate the internal spatial organization of sites and/or to determine the specific function of features, structures or spaces within sites”.

In this paper we present a pilot procedure to generate a ‘supplementary’ archaeological record where evident stratigraphy is lacking, survey conditions may be uneven and excavations limited, due to the overall site size. This record is aimed at creating horizontal archaeological signatures that trace activities not only inside and around the stone enclosed parts of the site, but of the spaces in between, which have the potential of enriching the understanding of the dynamic use of space. We propose herein the application of portable X-ray fluorescence analysis (pXRF) in a southern African SWS of historical age (18^th^ century CE) coupled with multivariate exploratory analysis based on geostatistical modelling. The aim of the paper is twofold: to explore the potential of the application of a low cost, quick, and non-destructive technique to detect chemical markers in anthropogenic sediments from a Stone Walled Site, and to envisage a way to analyse the results in order to improve our understanding of the use of space at non-generalized scales in such sites.

### The site of Seoke

The study site of Seoke in southeast Botswana has been the subject of archaeological research since 2012, within a project aimed at elucidating the dynamics of territorial expansion and identity construction of the Tswana-speaking group of the Bangwaketse from the late 1700s [16]. Originating c. 1700–1725 as a breakaway group from the Kwena, by 1780 the followers of Ngwaketse and his descendants had become a regional power. The Bangwaketse began their expansion during the rule of Moleta (c.1770-c.1790) when they also rose rapidly to control present southern Botswana. Through the work carried out by Morton and Merlo [43] that combined oral traditions, topocadastral information, survey and remote sensing, a number of archaeological settlements related to the age of Moleta have been recorded, including Seoke. According to oral histories collected in the 1920s and 1930s, the Ngwaketse capital of Seoke, where Moleta assumed power in c. 1770, was established during the reign of Makaba I followed by his son Mongala circa early to mid-1700s [44, 45].

The site is located in the freehold farm Woodlands 8JO presently known as “Lobatse Estates”, immediately northeast of Lobatse town.

The study area is underlain by several geological units that vary in age from Neoarchaean to Palaeoproterozoic (approximately 2.781 to 2.0 Ga; [46]). The Kanye Formation of the Lobatse Group, constitutes an intrusive homogenous felsite (which will give rise to highly silicious, sandy substrates). The intrusive felsites are unconformably overlain by younger (2.65 Ga) sedimentary rocks of the Black Reef Quartzite, represented by a sequence of dolomitic limestone, chert, minor limestone, ironstone, variably carbonaceous siltstone and shale ([46], Botswana Geoscience Portal, http://geoscienceportal.geosoft.com/Botswana/Search/). The Upper Transvaal Supergroup underlies the larger part of the study area, building the prominent topographic features including the hills to the east of the site. These 1.6 to 2.5 Ga aged metamorphic and sedimentary rocks collectively consist of inter-bedded reddish quartzite, shale, variably manganiferous and carbonaceous sandstone with chert, limestone, ironstone, andesitic volcanics and breccia ([46], Botswana Geoscience Portal). Geomorphologically, the study area is characterised by uniquely isolated hills with a maximum height of 350 m. No major rivers cut the study area, although large areas in the footslope and valley floor regions contain significant thicknesses of residual soil. The soils of our study area in particular are lithosols [47], and more specifically arenaceous sediments (i.e. with a sandy texture [48]). The bedrock is near the surface and weathered. Since the site is not on a steep slope, the dominant pedogenic factors are likely to be resistant parent material and a dry climate. The site area is characterized by shallow sands and loams. Soil drainage is fair to good, with a clay content of less than 15% [47:284]. The most common mineral is quartz (SiO_2_), although other minerals include feldspar, white mica and clay minerals. The mineral composition of wind-blown and alluvial sand in this region are observed to be dominated by quartz.

**Fig 2:**
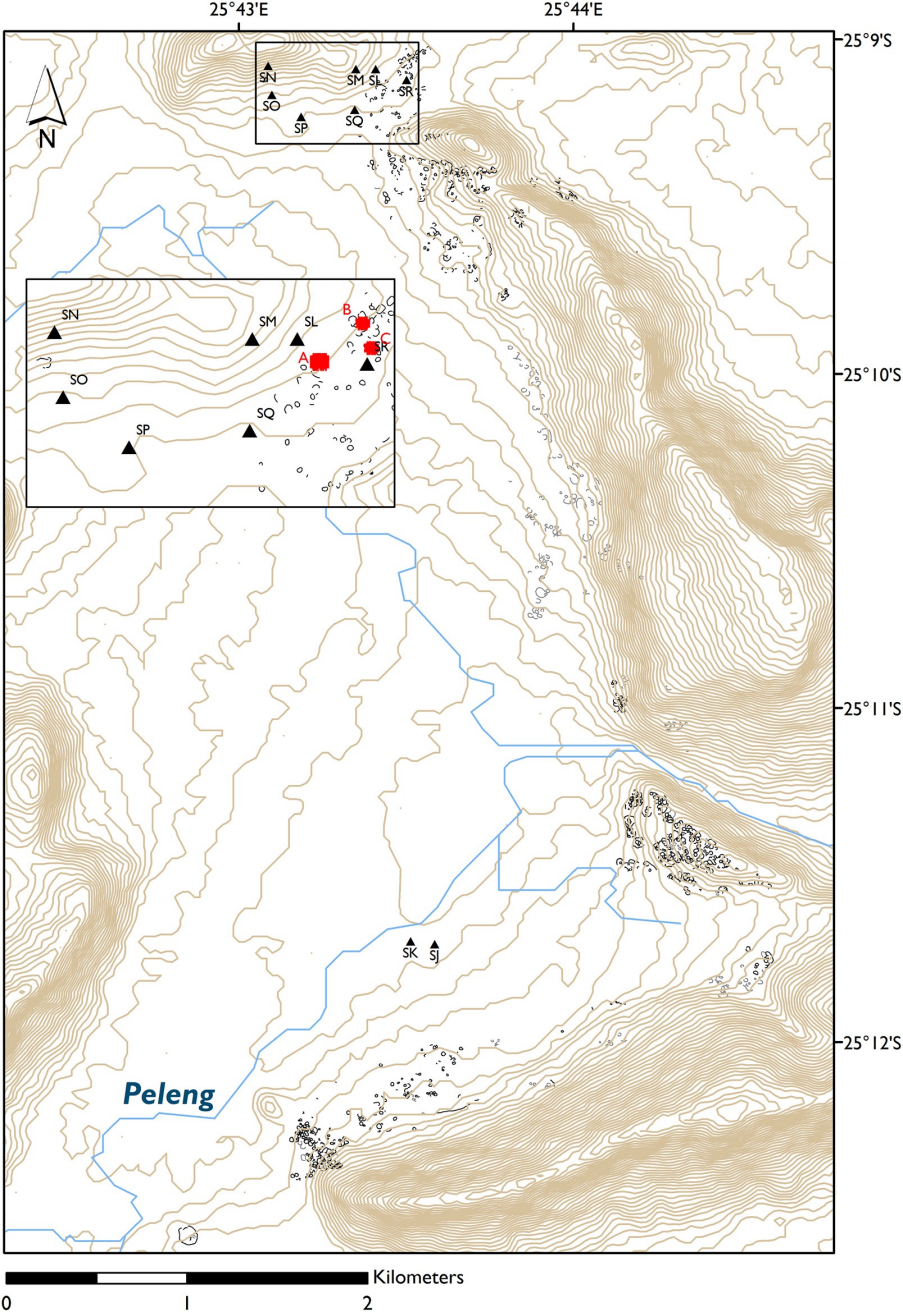
Map of Seoke SWS. Stone walls in black were surveyed on the ground, stone walls in grey were manually digitised by S. Merlo from GoogleEarth imagery and not verified on the ground. The position of the control samples (black triangles) and sampling areas (red squares) are indicated. Sources: contour data and rivers extracted from Aster DEM by S. Merlo.

**Fig 3:**
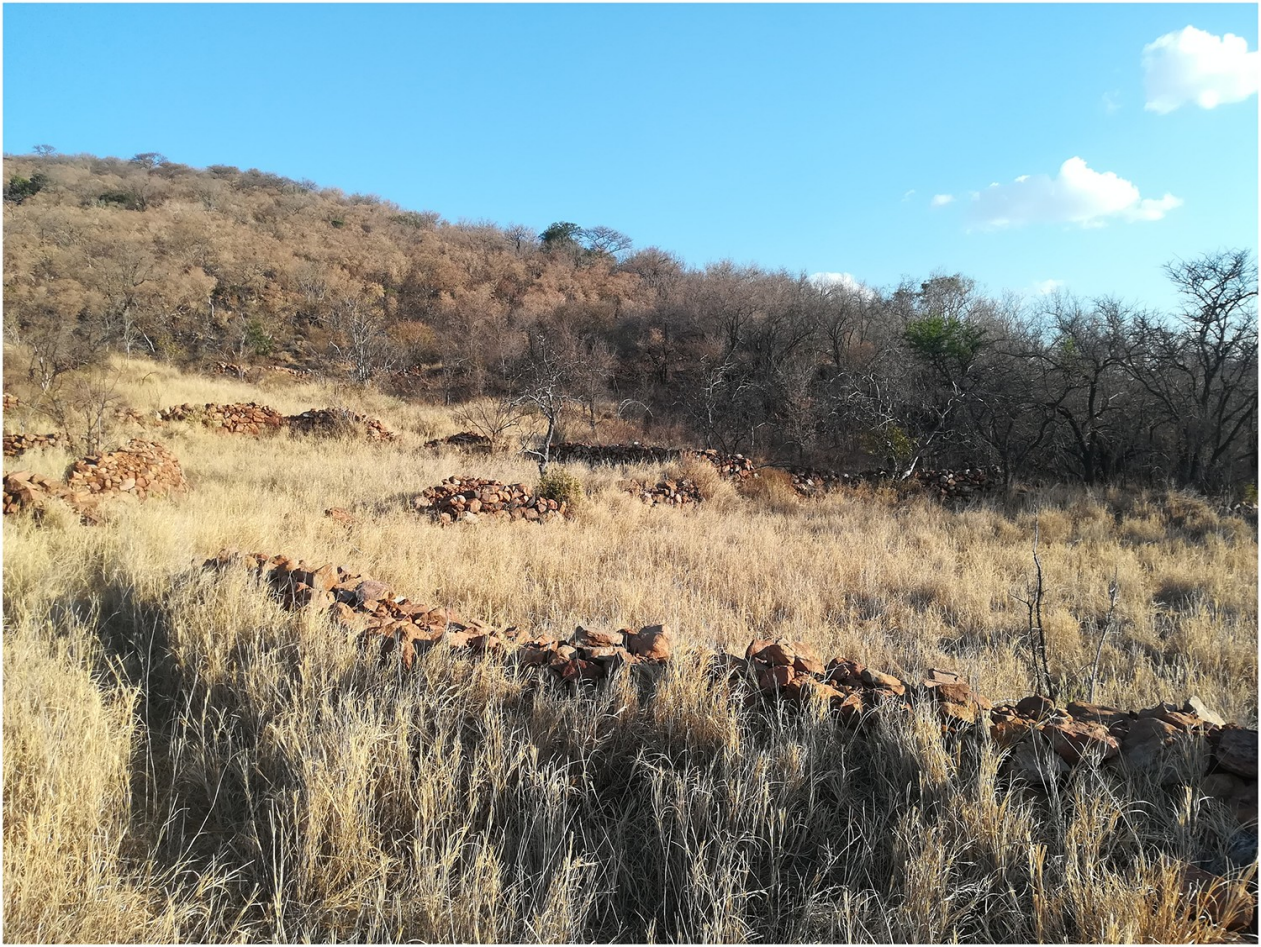
View of the site of Seoke, October 2019 Stone Walls at Seoke, southern cluster. *Cenchrus ciliaris* (buffalo grass) can reach 1,5 meters in height.

A combination of visual identification on GoogleEarth imagery, extensive handheld GPS survey and verification and detailed DGPS mapping of selected areas between November 2013 and October 2019 have revealed extensive stone walling in the area. The walling consists of separate but interlinked clusters of varying sizes and concentrations which extend along the lower slopes and at the foot of the hills, following the V- shaped alluvial plains of the Lobatse (Peleng) river over an area of 14.5 km^2^ (Fig 2), making it one of the most extensive CCP tradition, Late Iron Age, stone walled sites in Botswana and South Africa. Systematic foot survey, artefact recovery and excavation of 14 trenches of a standard size of 2x2 meters over selected middens and one iron smelting area have been carried out and are in the process of being published, alongside the analysis of the uncovered material remains. One of the main challenges of the archaeological work conducted at Seoke has been that of maximising information recovery in order to address the project’s archaeological questions and minimising the time and cost efforts of engaging an extensive area, particularly difficult to survey due to the presence of thick and almost impenetrable buffalo grass (*Cenchrus ciliaris*), which conceals the stone walling itself (Fig 3), let alone other elements of the landscape under investigation (middens, grain bins, ceramics, work areas, etc.). Systematic soil sampling and rapid geochemical analysis of soil offer a complementary and efficient alternative to intensive survey and excavation for mapping activities across large areas at sites such as the one described here. The method was applied to a portion of an area identified as the northernmost cluster, which remains the least affected by grass overgrowth, thanks to the presence of cattle grazing in the area. Systematic survey and the excavation of four 2x2 m trenches over 3 middens (excavated by level) and one food preparation area (excavated by context) were also carried out to corroborate the interpretation of the soil chemistry analytical results.

The site of Seoke is characterized, as other SWS, by a very thin and uneven archaeological deposit, generated in possibly c.30 years of continued occupation of different intensity throughout the site. While some middens feature up to 1m of archaeological buried deposit, the rest of the anthropogenic sediment throughout the site is characterized by an estimated thickness ranging between 5 and 20 cm (according to the experience of the authors in the field).

Although both oral historical accounts (which attest to the presence of the Kgwatheng) and sporadic archaeological surface finds attest to the occupation of this area prior to the arrival of the Bangwaketse, the stone walling at Seoke can by and large be associated with the Bangwaketse occupation (based on ceramics recovered on the surface in association with the stone walling). At Seoke, discrete clusters unified by coherent architectural features can be considered as ‘monophasic’. Their archaeological deposits are buried under a thin layer of sterile surface colluviums. After the abandonment by the Bangwaketse at the end of 1700s, the area where Seoke is located was intermittently used as cattle grazing land. As such, these deposits represent a unique opportunity to sample large and simultaneously occupied habitation surfaces.

### The use pXRF for the chemical characterization of anthropogenic sediments

The use of portable X-ray fluorescence devices (pXRF) is rapidly growing among those interested in performing fast and low-cost analysis of the chemical composition of sediments, soils, rocks, and artifacts. In the last decade, archaeologists too have discovered the advantages offered by handheld devices that can be easily transported to most remote locations. The non-destructive nature of pXRF and its capacity to provide quick results are obvious benefits for examining archaeological items that cannot be damaged or are not easily moved. In the study of ancient artefacts, pXRF has demonstrated its potential on potsherds, metals, lithics, and cuneiform tablets [e.g., 49–60]. The use of pXRF for archaeological sediments and soils, has also increased in the very last years. Janovski and colleagues [61] identified the chemical elements that relate to human activity in Tel Burna (southern Levant), shedding light on the formation processes of the archaeological deposit. Davis, MacFarlane and Henrickson 2012 [62] and Ginau et al. 2020 [63] focused on the analysis of archaeological profiles to analyze vertical variability in chemical elements in lithostratigraphic sequences and support chronological reconstruction. Holcomb and Karkansas [64] applied pXRF on resin-impregnated micromorphological block samples to complement their geoarchaeological study of the profile of an Archaic (7^th^ century BC) ritual ash midden from the site of Kalapodi (Greece).

pXRF is also being used for site prospection and for the characterization of activity areas in different archaeological contexts. Lubos and colleagues [42] compared the performance of pXRF analysis on soil samples with a wide range of techniques, and found that the current generation of pXRF is highly suitable for multielement analysis of archaeological sediments. Hayes and colleagues [65] showed that pXRF analyzer can be used to carry out surface geochemical survey on shallow sites, identifying some features’ fills. Frahm et al. [66] developed a method to measure P concentration in archaeological sediments and tested the results with ICP OES technique. Save and colleagues [32] have recently presented the results of large-scale surveys carried out with pXRF, stressing the effectiveness and high potential of this technique. Other case studies [e.g. 67–69] demonstrate the variety of application on archaeological sediment that pXRF is experiencing worldwide. Nevertheless, in spite of its rapidly growing use, the use of pXRF is not exempt of technical and methodological issues. Generic limitations in the use of pXRF are mainly related to accuracy, which depends on fewer detectable elements and lower sensitivity than other techniques, such as petrographic analysis, Neutron Activation Analysis, Inductively Coupled Plasma (ICP) Mass Spectrometry, ICP Atomic Emission Spectroscopy, Atomic Absorption Spectroscopy, Optical Emission Spectroscopy, Thermal Ionization Mass Spectrometry, and Stable Isotope Ratio Analysis. Moreover, low-Z elements such as for example Mg, S and Si, may present errors in detection as the pXRF does not generate a full vacuum and therefore air interferes with the quantification of such elements [49]. Due to its novelty as research tool in archaeology, voices for the elaboration of reliable systematic protocol to be readily applied by archaeologists have been repeatedly raised [70–73]. Our work presents a further case study on archaeological sediments and proposes the use of kriging as a method to map the results.

## Materials and methods

**Fig 4:**
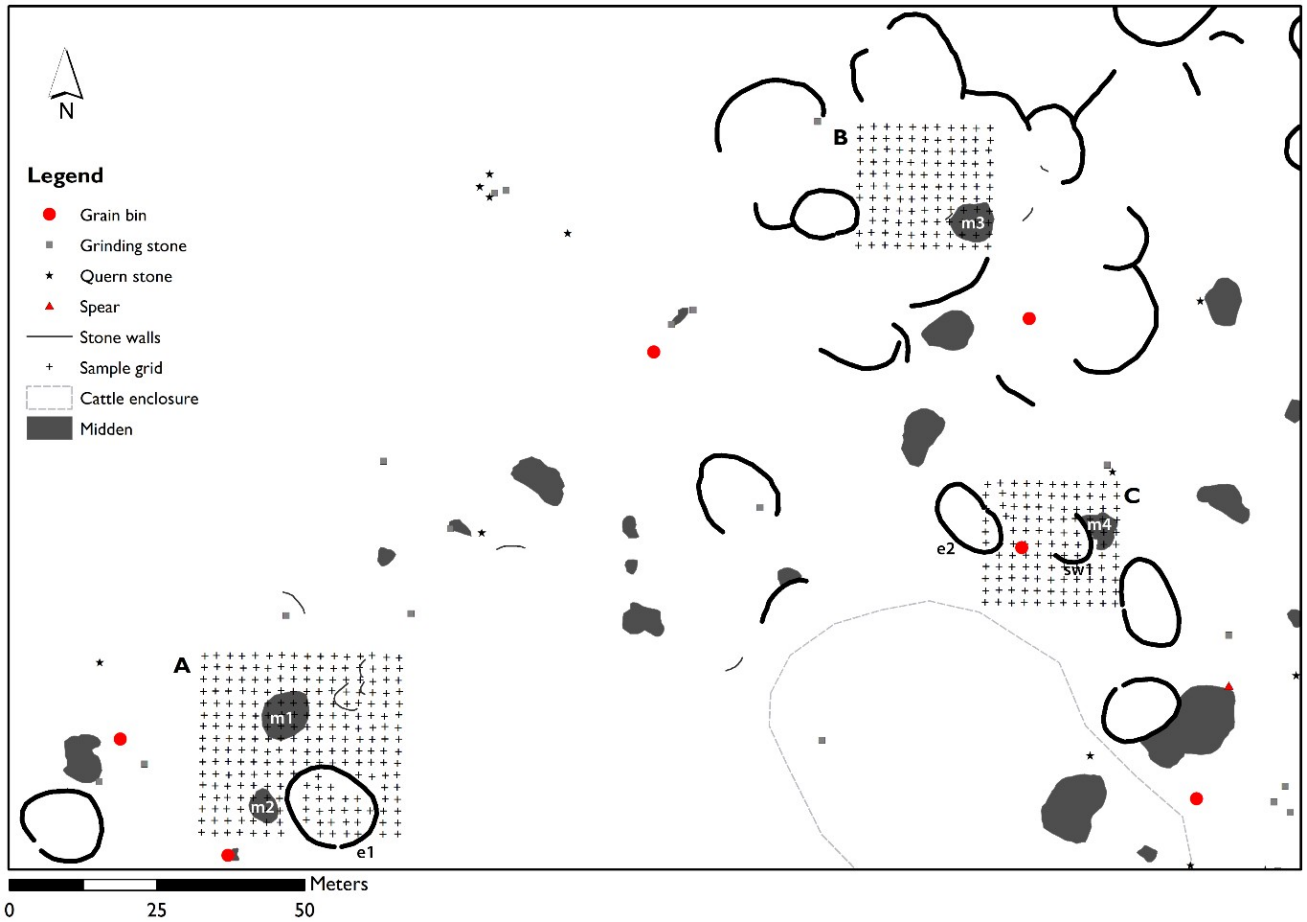
Map of the sampled areas at Seoke northernmost cluster showing detail of the features recognised during ground survey. The features discussed in the paper are numbered in a progressive way: enclosure 1 (e1), midden 1 (m1) and midden 2 (m2) in area A; midden 3 (m3) and enclosure 2 (e2) in area B; stone wall 1 (sw1) and midden 4 (m4) in area C.

The project and the field research were carried out under Government of Botswana research permit EWT 8/36/4 SSSVI(26), issued by the Ministry of Environment, Wildlife and Tourism, Gaborone. Three different areas of the northern part of the site were selected in relation to different archaeological features (Fig 4): area A (900 m^2^) with a circular cattle enclosure (enclosure 1) and two middens, one set north of the enclosure (midden 1) and one west of the enclosure (midden 2); area B (400 m^2^), the central area of a cluster of semicircular enclosures, including a midden (midden 3); and area C (400 m^2^), with a possible domestic feature (stone wall 1) with adjacent midden (midden 4) and a circular stone stock enclosure only partially sampled (enclosure 2). A grid of 2x2m was placed over each area. Ten grams of sediment were collected every 2 m following the grid, with the exception of a few points where stone walls were present. We sampled a total of 1500 m^2^ and collected 477 samples related to the archaeological deposit (i.e. within or close to visible stone structures) and 45 control samples. The last of these consisted of 4 to 5 sample points, located close to each other, distributed over 9 spots, in different parts of the study area (See Fig 2; see also S1 File for raw data). In order to check the instrument, 15 archaeological and 4 control samples were measured in triplicates: after checking that the measures were consistent these measures were averaged for the subsequent statistical analysis. Each sample was geolocated by means of DGPS and then carried to the store of the Seoke Project located in the Lobatse Estates (a few kilometers from the archaeological site), where pXRF analysis was carried out in a purposely set up field laboratory. Sediments were sieved, ground, and placed in plastic XRF cups covered by a thin polyester film and subjected to 120 seconds of analysis each, amounting to about 4 minutes per sample. A Thermo Niton Gold series pXRF was used, with Cu/Zn mining calibration. In this work we opted for the analysis of bulk samples rather than in-situ measurements so as to minimise the risk of skewed results due to irregularities of the sample and because of the extremely high temperatures at the site that quickly overheated the instrument. Indeed, the high outside temperature, especially at midday presented heating issues even in the sheltered space of the excavation house, and the analyses had to be intermittently stopped in order for the instrument to cool down. Soil samples were thus collected after a shallow cleansing of the surface consisting in the removal of c. 5 cm of surface sediment, to intercept the archaeological deposit buried under a thin layer of colluvium (see previous paragraph).

Chemical variables showing a high percentage (> 40%) of readings below the detection limit of the instrument were excluded from the analysis, as well as those elements presenting a Relative Standard Deviation (RSD) higher than 20%. Data were analysed using unweighted Logratio Analysis (LRA), a multivariate technique developed explicitly for compositional data and which is equivalent to apply Principal Component Analysis to the transformed Centred Logratio (CLR) variables [74]. Spatial interpolation of the elemental concentrations was performed by co-Kriging, using the Centred Logratio transformed variables for both structural analysis and prediction steps (extended with a residual component), and subsequently back transformed to obtain the actual concentrations (in %). Empirical variograms were computed using a maximum distance of 20 m between data pairs (with a total of ten lag bins). Based on the examination of the variograms, we adopted a linear model of coregionalization comprising three basic structures: a nugget effect, a short range spherical model (range = 7 m), and a large range exponential model (range = 10 m). The model fitting has been performed by a weighted least squares approximation, using the iterative algorithm proposed by Goulard and Voltz [75] and a weighting scheme consisting in the number of data pairs within each lag bin. Elemental concentrations have been interpolated using the samples of each area separately.

The R code used for analysis together with the datasets can be found in GitHub (https://github.com/cl379/papers_supl_materials/tree/master/Biagetti2020_Seoke).

## Results

Table 1 presents the summary statistics of absolute values of the elements analysed (the full dataset of the raw readings from the instruments can be found in S1 File).

**Table 1:** Summary statistics of the raw values for archaeological and control samples. Correlation coefficients can be found in S2 File.

		Al	Si	P	S	Cl	K	Ca	Ti	Cr	Mn	Fe	Zr
Archaeological	Min.	0.097	2.236	0.423	0.052	0.051	0.423	0.327	0.179	0.002	0.027	1.862	0.018
	1stQu.	0.143	3.479	0.762	0.088	0.058	0.680	0.497	0.243	0.007	0.069	2.311	0.026
	Median	0.161	3.670	0.793	0.094	0.060	0.749	0.627	0.260	0.008	0.083	2.478	0.029
	Mean	0.1675	3.678	0.785	0.093	0.061	0.763	0.876	0.259	0.008	0.088	2.482	0.029
	3rd Qu.	0.186	3.869	0.815	0.099	0.062	0.828	0.897	0.274	0.009	0.098	2.651	0.032
	Max.	0.557	6.242	0.973	0.153	0.233	1.156	8.772	0.326	0.014	0.230	3.154	0.045
Control	Min.	0.131	2.770	0.077	0.048	0.044	0.076	0.075	0.034	0.002	0.005	0.385	0.002
	1stQu.	0.177	3.645	0.640	0.085	0.060	0.458	0.381	0.236	0.008	0.087	2.383	0.022
	Median	0.211	4.023	0.697	0.091	0.062	0.591	0.463	0.261	0.009	0.237	2.736	0.027
	Mean	0.754	7.477	0.647	0.164	0.067	0.627	0.999	0.254	0.011	0.457	4.401	0.025
	3rd Qu.	0.258	4.366	0.744	0.099	0.065	0.732	0.622	0.276	0.013	0.619	3.469	0.031
	Max.	6.928	41.625	0.898	0.825	0.156	1.304	19.914	0.427	0.038	2.166	30.246	0.043

**Fig 5:**
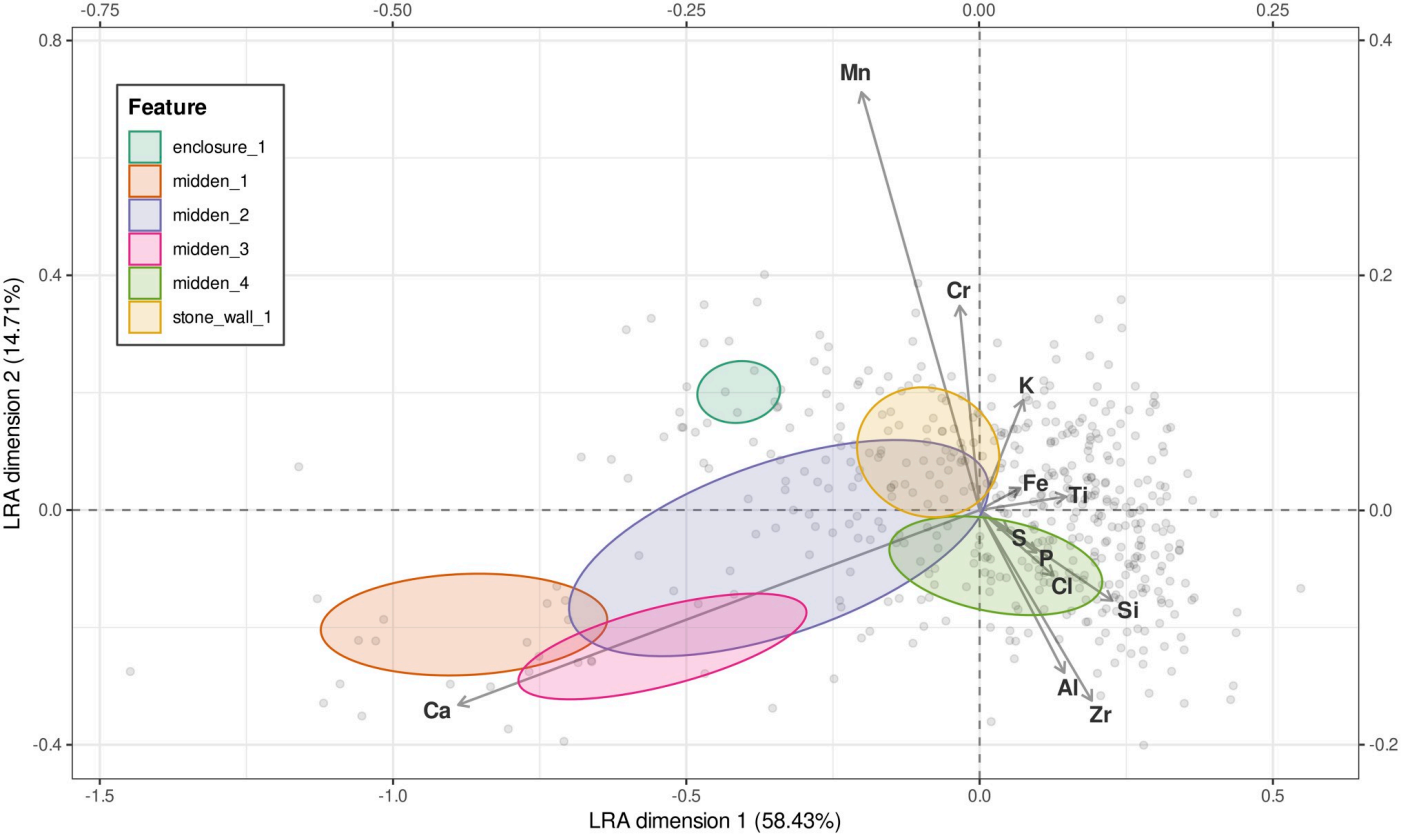
Unweighted Logratio Analysis (LRA) biplot of samples, with 95% confidence ellipses for the feature types (enclosure 2 has been excluded).

**Fig 6:**
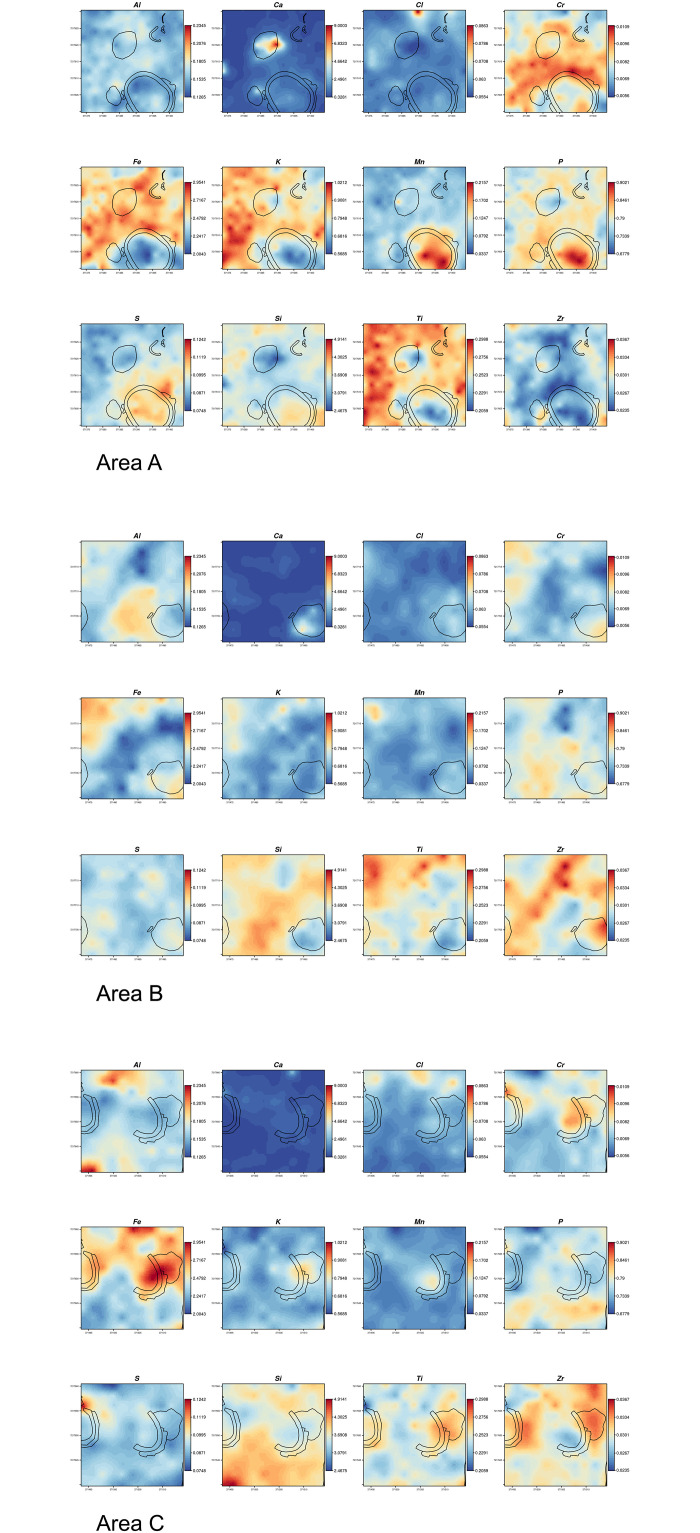
Co-Kriging map of elemental concentration at Seoke at areas A-C. Values express absolute concentrations (in %).

A first exploration of the data on samples assigned to a category according to their position in space, revealed the presence of groupings (Fig 5, LRA scores are provided in the S3 File). Although the variance explained by the first two principal components is not very high (45%), it can be observed that there is a certain degree of separation between area A and the other two areas. Within area A, the stock enclosure samples are clearly separated from the rest, with midden 1 samples, and midden 2 samples being different. These last samples tend to overlap with the midden in area B, whereas the midden samples in area C form a separate group. The majority of the samples from the enclosure in area C are almost totally unrelated to the enclosure samples of area A. Finally, the samples from the stone structure in Area B seem to be distributed across all groups. The rest of the samples not directly associated with an activity area or structure display a high variance across both LRA dimensions. The results of co-Kriging analysis are shown in Fig 6.

As evidenced by both the co-Kriging (Fig 6) and the LRA (Fig 5), the two enclosures present enrichments in different components. This result, however, is influenced by the limited number of samples analysed for the enclosure 2 (n = 4) and their position within the structure, all very close to the wall. Enclosure 1 is especially enriched in Mn and P and moderately enriched in S, Si and Cr. Considering the nature of this structure, it is not surprising that P is up to 2.3 times higher than the average of samples. Midden 1 and midden 3 present very similar enrichments, especially relevant for Ca; midden 2 also presents a similar composition though the enrichment is less marked. Middens 1 and 3 present depleted values of Si whereas Midden 2 and 4 display average values of Si. Stone wall 1 is very different from all the middens and is characterised mainly by elevated values of Mn and Fe.

The areas between the stone structures present relevant enrichments in specific elements:

- Silicon: relevant enrichments of Si are located in the SE corner of area A (outside the enclosure), and in the southern half of area B, where it features a peculiar “inverted V” shape, whose apex falls where a grain bin has been recorded, and also throughout area C;
- Chlorine: an enriched spot is located in the northern part of areas A;
- Potassium: this element is very high in area A especially to the W of the middens, as well as in the NW corner of area B. Some enrichment in midden 3.
- Titanium: this heavy metal presents similar enrichment patterns as K.
- Iron: enriched spots of Fe are found in area A especially to the W of middens, as well as in the NW corner of area B and, in addition, in the northern part of area C.
- Zirconium: presents high values between the structures of area B.

**Fig 7:**
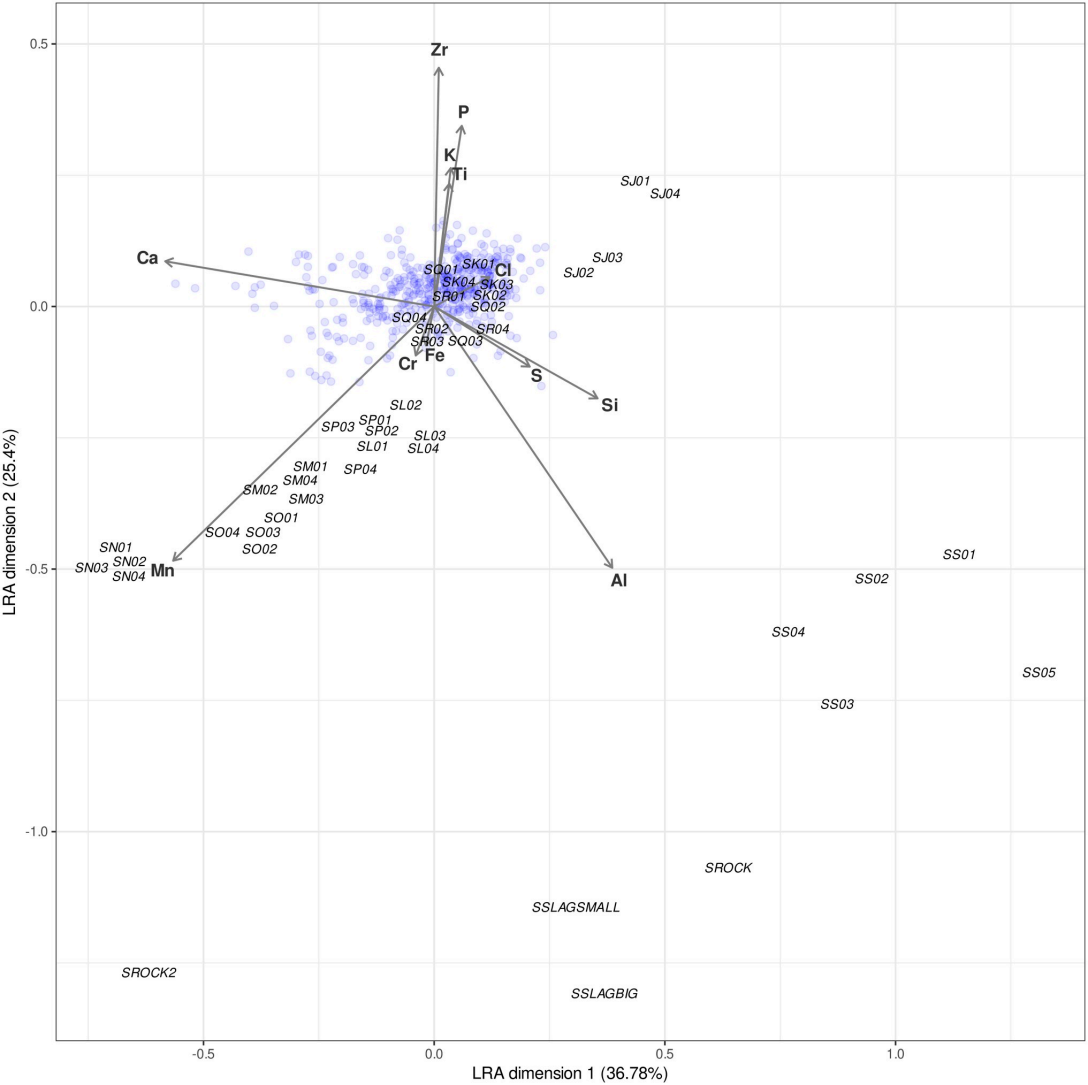
Logratio Analysis (LRA) including the control samples (labeled). Blue dots correspond to the “archaeological” samples.

Samples outside of the three areas (A-C) have been collected from different points in the site (Fig 2). Fig 7 shows the LRA including these control samples. Individual samples from the eight areas taken as a control generally group quite closely together, indicating that the sampled areas were quite internally homogeneous. Samples SK and SJ were the furthest from the visible structures, the former located along the river floodplain, and the latter on a small rocky outcrop, and while SK clusters are set very far from the archaeological samples, SJ points are positioned right at the edge of the archaeological cloud. Points SL, SP, SQ and SR fall completely within the archaeological samples cloud: SL, SQ, and SR were collected not far from visible archaeological structures; SP sample points however, were located in an area that is supposedly outside the archaeological site. SM and SO, similarly to SJ, cluster at the edge of the archaeological cloud points. SN -collected on the opposite side of the hills in respect to the archaeological structures- and SS -collected within site but right on top of rocky outcrops- fall completely outside the archaeological cloud, as do the slag and rock samples.

## Discussion

The pXRF analysis carried out at Seoke has recorded the most diagnostic elements related to human occupation, including Ca, K, P and Mn. Those elements in particular are associated with a number of daily activities [32, 41] and hint at past use of space in our site. Looking at the results of the spatial distribution of the various elements, together with the archaeological features visible on the surface, hypothetical inferences can be made regarding the use of space at Seoke.

Phosphorus (P) is rather widespread throughout the three sampled areas, testifying to the presence of cattle roaming throughout the site. Detection of P by pXRFs has been questioned [49] although fresh research suggests that recent instruments can quantify P even at low concentrations in archaeological soil and sediments [66]. The average value of P in the different areas analysed (S1 File) is virtually the same (enclosure 1: 0,83; enclosure 2: 0,75; midden 1: 0,77; midden 2: 0,79; midden 3: 0,78; midden 4: 0,79; stone wall 1: 0,76; undetermined: 0,78). Nevertheless, our geostatistical analysis clearly shows that the stock enclosure in area A (enclosure 1) displays high enrichment of P, along with Mn, S, and Si, highlighting the role of co-Kriging in pinpointing anomalies in the distribution of chemical elements in anthropic deposits that contribute to the reconstruction of the use of space. Considering the nature of this structure, it is not surprising that P is up to 2.3 times higher than the average of samples (Fig 6). Those elements -P in particular- have been correlated in previous studies with animal dung or enclosures [76, 77]. Regarding enclosure 2, only one sample seems to confirm its use as a livestock enclosure (similar to enclosure 1), while the other 3 samples fall within the main cluster of points (Fig 5). Further samples may clarify the very use of this structure.

Anomalies in the distribution of chemical elements confirm that the areas that were identified as middens during the foot survey, actually present different chemical signatures from the surrounding areas. Middens in Tswana settlements have been discussed in a number of papers and their excavation represents a fundamental approach to the study of past Tswana towns. Different types of middens have been recognised in Tswana settlements according to their location, size and use, ranging from *kgotla* (central court) middens, to communal dumps (located in front of a series of households and in intervening areas between stone walls) to single household discard areas related to a nearby structure and generally located immediately behind a courtyard wall [78]. Communal middens, located in a visible area, are more likely to contain ashes and organic materials, which are believed to be a medium for witchcraft in Bantu ethnography [79, 80], compared to individual household middens, which can be less easily controlled and are therefore less likely used for such materials. The middens located in area A and B (midden 1, 2, 3)—most likely communal middens—feature similar enrichments likely related to the disposal of organic waste, while the midden located in area C behind a wall (single household midden -midden 4) presents a different chemical signature not strictly connected to organic matter. The enrichment in metals (Zr, Fe, Mn, and Cr) recorded throughout the whole area C may result from some activity related to metal performed in front of the semicircular stone wall. The occurrence of Zirconium in midden 4 in area C has been linked to the presence of pottery, Zr being a common component of clay. Recent research has correlated enrichments of Zr to areas of vessel storage [32]. In our area C, the chemical elements recorded may fit with the hypothesis of an area used as a ‘workshop’ or storage, where metal tools may have been used for shaping pottery, clearing, breaking wood for fires, trimming plaster (cow dung and mud) walls, as well as sharpening them on stones.

Notwithstanding the issues related to the measurement of Si in absence of a vacuum, this element presents interesting patterns, especially considering that this type of error should be constant throughout the measures and therefore be of less impact when looking at relative distributions. Indeed, although the substrate is quite rich in silicon in general, the strong enrichment of this element identified in area C is in spatial connection with the base of a grain bin. This might indicate that cereals were processed in this area and thus, the silicon enrichment could be produced by the higher presence int his spot of crop processing leftovers and in particular, of phytoliths (silica bodies produced by plants which are particularly abundant in grasses and cereals). If this was the case, activities connected to cereal processing and storage could therefore be hypothesized in at least two areas in the site of Seoke: the space between the structures in area C and in area B. In addition, the moderate enrichment of Si visible in the enclosure 1 (area A) could be interpreted as the signature of phytoliths in livestock faecal remains [76, 81, 82]. The presence of Chlorine (Cl) in some parts of area A and C, and particularly in a spot set in the northernmost edge of area A, is hardly interpretable in our case and can be an artefact even though, based on the geology and climate of the area, it is unlikely that significant Cl can be naturally added to the soil from the bedrock or through water accumulation. Within the three sampled areas (A to C), multielement chemical analysis has therefore outlined chemical differences in the soil composition throughout the site that (i) confirmed prior interpretation of archaeological features such as middens and a stock enclosure, (ii) may have detected activity areas that otherwise are not visible by simple visual inspection of the site. An example of the latter is represented by a spot visible in the upper left corner of area B, whose enrichments in Fe, Mn, and Ti hints at some anthropic activity performed there that requires further sampling to be fully understood.

Control samples collected outside of the three areas (A to C) provide further insights on the size of the archaeological site and the land use of the whole area. A floodplain used as a grazing area in present times (control sample SK), presents significantly different signatures (see Fig 7) from the archaeological site, helping in characterizing the archaeological chemical signal. In a region where cattle have been traditionally left free to roam (including within archaeological sites), the contamination of the thin and exposed archaeological layer by animal droppings through time could have occurred. Control samples were taken far from the site (Fig 2). The results of the LRA in Fig 7 show how the control samples distribute against the cluster generated by the samples collected from areas A to C (Fig 7). The spatial distance of the points where control samples were taken is reflected in the spatial distribution in the LRA (Fig 7). Our study therefore allows us to distinguish between the site area and non-site area due to the difference in the distribution of chemical elements, and helps in recognizing the limits of the area occupied in the past, thus outlining the edges of the archaeological site. This is key to identifying the site area beyond the presence of stone walls. The exceptions are samples SP and SQ that cluster with the archaeological samples. In this case, further survey and sampling is necessary to ascertain whether SP and SQ represent off-site samples or rather these have been collected from an area occupied in the past and, so far, not recognized and included in the ‘site’.

## Conclusions

The use of non-invasive techniques is opening unprecedented possibilities into the understanding of African archaeological sites, without disturbing the cultural heritage with new excavations [83]. In this study we have explored the potential of pXRF combined with geostatistics to understand the use of space beyond the visible archaeological evidence. Our study has provided insight on the utilization of space, confirming or shedding light on the possible functions of sampled areas. This research has also pinpointed the existence of other features that were not recognized in the field. If applied to larger surfaces, our methodology promises to expand and support the archaeological interpretation of ancient and historic settlements. The most promising achievement of our research is that pXRF performs well in Stone Walled Sites and, although much prospecting is needed, the results herein presented can be critically used to design surveys and excavations in other Stone Walled Sites, and, more generally, on open air sites.

The use of pXRF at Stone Walled sites represent a quick and effective way to approach such a peculiar archaeological record, overcoming the issue of its exceptional thinness. pXRF provides quick results, since no longer than four minutes per sample is needed, including sieving and grinding, allowing to analyse relatively large areas in a very short time. The field lab can be easily set up in the house or shelter and avoid the hassle of transporting large quantities of bulk sediment to a laboratory elsewhere, sometimes with complicated export permit procedures. Doubtlessly, our technique needs to be tested in other sites and combined such archaeological proxies, as phytoliths, organic residues and the characteristics of the visible archaeological evidence (stone walls). In spite of the very young age of the Seoke archaeological deposit, post-depositional processes might have affected the distribution of chemical elements. Further taphonomical assessment, coupled with more geoarchaeological investigatigation (e.g. thin sections from identified features, study of other micro-proxies such as organic residues or phytoliths) is certainly required, in order to shed light on possible alterations and disturbances to the deposit. Generally speaking, the use of pXRF on anthropogenic sediments is still at an early stage, and further studies are needed to properly refine this procedure.

## Supporting information

S1 File Raw data and summary statistics. This spreadsheet contains 4 tabs for raw data of pXRF measurements for all samples and summary statistics for archaeological samples, control samples and samples by group.
(CSV)

S2 File Correlation coefficients. (CSV)

S3 File LRA scores. (CSV)
