# Correction: Identifying anthropogenic features at Seoke (Botswana) using pXRF: Expanding the record of southern African Stone Walled Sites

**DOI:** 10.1371/journal.pone.0269051

**Published:** 2022-05-23

**Authors:** Stefano Biagetti, Jonas Alcaina-Mateos, Abel Ruiz-Giralt, Carla Lancelotti, Patricia Groenewald, Jordi Ibañez-Insa, Shira Gur-Arieh, Fred Morton, Stefania Merlo

The seventh author’s name is spelled incorrectly. The correct name is: Shira Gur-Arieh. There is also an error in affiliation 6 for author Shira Gur-Arieh. The correct affiliation 6 is: The Leon Recanati Institute for Maritime Studies, University of Haifa, Haifa, Israel.
